# Editorial: Signaling Control by Compartmentalization Along the Endocytic Route

**DOI:** 10.3389/fcell.2019.00237

**Published:** 2019-10-17

**Authors:** Costin N. Antonescu, Allen P. Liu

**Affiliations:** ^1^The Graduate Program in Molecular Science, Department of Chemistry and Biology, Ryerson University, Toronto, ON, Canada; ^2^Keenan Research Centre for Biomedical Science of St. Michael's Hospital, Toronto, ON, Canada; ^3^Department of Mechanical Engineering, University of Michigan, Ann Arbor, MI, United States; ^4^Department of Biomedical Engineering, University of Michigan, Ann Arbor, MI, United States; ^5^Cellular and Molecular Biology Program, University of Michigan, Ann Arbor, MI, United States; ^6^Biophysics Program, University of Michigan, Ann Arbor, MI, United States

**Keywords:** endocytosis, signaling, compartmentalization, GPCR, receptor tyrosine kinase

Many different receptor systems are regulated by endomembrane traffic, including signals from receptors activated by extracellular cues such as receptor tyrosine kinases (RTKs), G protein-coupled receptors (GPCRs), morphogen receptors, and immune receptors. Notably, with few exceptions, each receptor system undergoes internalization from the cell surface upon activation, followed by receptor-specific control of intracellular membrane traffic through complex intracellular endosomal compartments. The canonical view of regulation of receptor signaling by endocytosis involves downregulation of receptors by their sequestration from the extracellular milieu rich in ligands or degradation of receptor-ligand complexes following traffic to the lysosome. However, it has become apparent that a wide variety of receptors and their downstream signals exhibit actions unique to specific endomembrane compartments (Sorkin and von Zastrow, 2009; Platta and Stenmark, 2011; Sigismund et al., 2012; Di Fiore and von Zastrow, 2014). Moreover, many of the membrane compartments of the endocytic system act as platforms for integration of signals that initiate at the cell surface from extracellular cues with signals derived from internal, cell-autonomous cues, such as metabolic or damage signals. As such, regulated endocytosis and membrane traffic define not only the duration and magnitude of signaling, but also allows activation of distinct signaling events in a spatially defined manner as well as context-specific regulation of signaling, thus specifying unique cellular outcomes. This Research Topic includes several review articles of important concepts related to how endocytic traffic and compartments function to regulate a variety of signals, as well as some primary studies that reveal novel aspects of regulation of cellular signaling by endocytic traffic and compartmentalization.

The role of ubiquitinylation in promoting the lysosomal degradation of signaling receptors has been well-documented. Focusing on GPCRs, Burton and Grimsey examine emerging novel regulation and functions of receptor ubiquitination. First, this review examines the evidence for possible compartment-specific regulation of GPCR ubiquitination, including that of PAR1. Moreover, this review examines how ubiquitination of PAR1 and other GPCRs by the E3 ubiquitin ligase NEDD4 controls receptor signaling leading to atypical activation of p38 mitogen activated protein kinase (MAPK), a phenomenon which may require endosomal recruitment of receptor and signaling intermediates. In addition, the potential for pharmacological intervention of putative endosomal signals activated by PAR1 and other GPCRs that trigger p38 MAPK activation for treatment of patients with intracranial hemorrhage and other injuries is highlighted.

mTOR (mechanistic target of rapamycin) signaling that regulates cell growth and proliferation has been classically associated with the lysosomes. Under mechanical stimulation, Lin and Liu reported that mTOR in skeletal muscle is recruited to and activated at macropinosomes. Mechanical stretch and osmotic shock induced activation of phospholipase D2, but not D1, and stimulated phosphatidic acid production on macropinosomes, leading to mTOR signaling. The surprising finding that mTOR can be activated in macropinosomes, in addition to lysosomes, underscores the complexity of the spatiotemporal dynamics of mTOR signaling that has implication in development and disease progression.

Lysosomes serve the critical function of effecting degradation of macromolecules within cells; however, these organelles are becoming well-appreciated as having far more complex functions that impact many aspects of cellular physiology. Inapanathan and Botelho examine how lysosomes serve as signaling hubs, functioning as platforms for recruitment and regulation of important signaling proteins such as AMP-activated protein kinase (AMPK), mTORC complex 1 (mTORC1), and glycogen synthase kinase 3ß (GSK3ß). This review examines how the highly regulated localization of each of these signals to the surface of the lysosome allows integration of cues such as from cell metabolism, extracellular mitogens, and immune signals into a coordinated cellular response. The review further discusses how these lysosome-derived signals in turn control autophagy, lysosome adaptation, cell proliferation and survival and immune cell function and many other outcomes, and how these observations support the view of lysosomes as a major signaling hub of the endocytic system.

The interplay between plasma membrane organization and vesicular trafficking and signaling is nicely illustrated in the context of immunological synapse (IS), a specialized contact area between a T cell and an antigen presenting cell. Onnis and Baldari reviewed a body of literature that points to trafficking pathways that deliver endosomal T-cell antigen receptor (TCRs), kinases, and adapter proteins to the IS in order to sustain signaling. TCRs can be internalized by both clathrin-dependent or clathrin-independent pathways, in a ligand-dependent or independent manner. Although classically known for signal termination, TCR endocytosis is followed by distinct polarized recycling pathways that contributes to IS assembly. Thus, paradoxically, TCR endocytosis is important for both signal termination and sustained signaling. The exocytic trafficking at the IS is also believed to be involved in intercellular communication to modulate antigen presenting cell function.

The endocytic system is intimately linked with signaling from RTKs, in particular for signals involving phosphatidylinositol-3-kinase (PI3K) and Akt. In Sugiyama et al., we examine the compartmentalization of PI3K and Akt activation, regulation and signaling to downstream substrates on various membrane compartments including the plasma membrane, endosomes, lysosomes, and mitochondria, as well as within nanoscale domains within compartments such as the plasma membrane. A particular focus is the recruitment of PI3K-Akt signals and regulators to the plasma membrane and endocytic vesicles derived therefrom, as this may suggest a mechanism for spatiotemporal coordination of positive and negative regulators of this signaling axis. Furthermore, this review examines how the compartmentalization of these key signals at various scales within the endocytic network impacts cellular processes such as invadopodia formation, metabolism, and lysosomal function.

The interplay between receptor internalization and signaling is expected to take place during directed cell migration process such chemotaxis. In DeNies et al., we investigated the relationship between clathrin-mediated receptor internalization and signaling for a chemokine receptor CXCR4. As expected, CXCR4 internalization is significantly reduced when clathrin is knocked down. Interestingly, we reported an increase in chemokine CXCL12-dependent ERK1/2 signaling but a decrease in cell migration under clathrin-knock down condition. However, disrupting lipid raft and CME simultaneously abrogated ERK1/2 signaling. An antibody sensitive to CXCR4 post-translational modification (PTM) revealed a decrease in CXCR4 PTM upon clathrin knock down, despite an unchanged total CXCR4 protein and mRNA levels. The study highlights the growing understanding that modulation of endocytosis affects not only receptor signaling and internalization but also receptor PTM.

Altogether, these studies and topical reviews underline the importance and regulation of signal compartmentalization at different cellular locales. Important unanswered questions remain, such as exactly how intracellular pools of receptors communicate with cell surface activated receptors and extracellular ligands. Could internal receptors be post-translationally modified in the absence of ligand stimulation? If so, what might be the physiological relevance of such signal compartmentalization? What are the unique physiochemical properties of specific endocytic compartments that endow these with unique signaling factors, and what is the specific repertoire of signaling factors in each endocytic compartment? Systems biology of receptor trafficking and signaling approaches will be important to unravel the mystery of spatial compartmentalization of receptor signaling. Finally, how receptor crosstalk plays a role in signaling compartmentalization, and how alterations of endocytic traffic can contribute to specific diseases by driving changes in receptor signaling will also be important future research directions.

## Author Contributions

CA and AL both conceived and wrote the editorial.

### Conflict of Interest

The authors declare that the research was conducted in the absence of any commercial or financial relationships that could be construed as a potential conflict of interest.
